# Targeting Morphine-Responsive Neurons: Generation of a Knock-In Mouse Line Expressing Cre Recombinase from the Mu-Opioid Receptor Gene Locus

**DOI:** 10.1523/ENEURO.0433-19.2020

**Published:** 2020-05-29

**Authors:** Julie Bailly, Natalie Del Rossi, Léonie Runtz, Jing-Jing Li, DaWoon Park, Grégory Scherrer, Arnaud Tanti, Marie-Christine Birling, Emmanuel Darcq, Brigitte L. Kieffer

**Affiliations:** 1 Department of Psychiatry, McGill University, Douglas Hospital Research Centre, Montreal, Quebec H4H 1R3, Canada; 2 Department of Cell Biology and Physiology, UNC Neuroscience Center, The University of North Carolina at Chapel Hill, Chapel Hill, NC 27599, USA; 3 New York Stem Cell Foundation – Robertson Investigator; 4 CELPHEDIA, PHENOMIN, Institut Clinique de la Souris (ICS), 67404 Illkirch-Graffenstaden, France

**Keywords:** cell-specific gene targeting, Cre-loxP system, enkephalins, knock-in mice, morphine, Oprm1 gene

## Abstract

The mu-opioid receptor (MOR) modulates nociceptive pathways and reward processing, and mediates the strong analgesic and addictive properties of both medicinal as well as abused opioid drugs. MOR function has been extensively studied, and tools to manipulate or visualize the receptor protein are available. However, circuit mechanisms underlying MOR-mediated effects are less known, because genetic access to MOR-expressing neurons is lacking. Here we report the generation of a knock-in *Oprm1*-Cre mouse line, which allows targeting and manipulating MOR opioid-responsive neurons. A cDNA encoding a T2A cleavable peptide and Cre recombinase fused to enhanced green fluorescent protein (EGFP/Cre) was inserted downstream of the *Oprm1* gene sequence. The resulting *Oprm1-*Cre line shows intact *Oprm1* gene transcription. MOR and EGFP/Cre proteins are coexpressed in the same neurons, and localized in cytoplasmic and nuclear compartments, respectively. MOR signaling is unaltered, demonstrated by maintained DAMGO-induced G-protein activation, and *in vivo* MOR function is preserved as indicated by normal morphine-induced analgesia, hyperlocomotion, and sensitization. The Cre recombinase efficiently drives the expression of Cre-dependent reporter genes, shown by local virally mediated expression in the medial habenula and brain-wide fluorescence on breeding with tdTomato reporter mice, the latter showing a distribution patterns typical of MOR expression. Finally, we demonstrate that optogenetic activation of MOR neurons in the ventral tegmental area of *Oprm1*-Cre mice evokes strong avoidance behavior, as anticipated from the literature. The *Oprm1*-Cre line is therefore an excellent tool for both mapping and functional studies of MOR-positive neurons, and will be of broad interest for opioid, pain, and addiction research.

## Significance Statement

Here we develop an innovative tool to characterize circuit mechanisms underlying opioid actions, which may help the research communities to improve the knowledge on circuitry adaptation and response to opioids. The tool is particularly relevant in the context of the current opioid crisis. Medicinal and abused opioids act primarily on mu-opioid receptor (MOR), and we developed here a Cre mouse line to specifically target and manipulate MOR-expressing neurons. This resource has huge potential for mapping, molecular characterization, and functional studies of opioid-responsive neurons.

## Introduction

The mu-opioid receptor (MOR) is the primary molecular target for medicinal and abused opioids. This receptor mediates both the unrivaled analgesic properties of opioids for pain treatment, and their adverse effects, including notably their strong addictive potential (Matthes et al., 1996; Darcq and Kieffer, 2018), which is driving the current opioid epidemic (World Health Organization, 2017; Volkow and Koroshetz, 2019). Under physiological conditions, MOR is activated by endogenous opioid peptides, and modulates nociceptive pathways (Corder et al., 2018), respiration centers (Levitt and Williams, 2018), and brain circuits that process reward and emotions (Contet et al., 2004; Lutz and Kieffer, 2013). Although the essential role of MOR in pain, drug abuse, and mood disorders is well established, and receptor adaptations to chronic opioid use have been well studied at the cellular level (Williams et al., 2013; Cahill et al., 2016), circuit mechanisms underlying MOR function (Darcq and Kieffer, 2018) and the regulation of neuronal communication driven by MOR (Mechling et al., 2016) and MOR agonists (Nasseef et al., 2019) are poorly understood.

Several genetic mouse tools have been developed to study MOR function, but have not given genetic access to MOR-expressing neurons as yet. A knock-in MOR-mCherry mouse line was developed to map MOR protein expression throughout the nervous system brain (Gardon et al., 2014; Erbs et al., 2015), and it allows speculation about the circuit mechanisms driving MOR-mediated behaviors. Mice with a floxed *Oprm1* gene have permitted receptor deletion in targeted neurons from nociceptive (Weibel et al., 2013) and reward (Charbogne et al., 2017) pathways, uncovering some circuit mechanisms of MOR-mediated pain control and motivation. A next step to understand MOR physiology, and to fully investigate neural dysfunctions associated with opioid drug use, misuse, and abuse, is to study and manipulate the activity of MOR-expressing neurons that directly respond to both exogenous and endogenous mu-opioids.

To this aim, the best approach is to create a mouse line expressing the Cre recombinase in MOR-expressing neurons. Here we report the generation of a line expressing the Cre recombinase under the control of the *Oprm1* gene (encoding MOR) promoter, and present molecular and behavioral characterization of this mouse line (*Oprm1*-Cre line). We also show successful labeling of MOR-positive neurons using a fluorescent Cre-dependent reporter mouse line. We finally demonstrate that optogenetic stimulation of MOR-positive neurons in the ventral tegmental area is sufficient to induce strong avoidance behavior, as anticipated from the literature. The line fulfills all the criteria to successfully study and manipulate MOR neurons.

## Materials and Methods

### 

#### Animals

The *Oprm1*-Cre knock-in mouse line was generated by homologous recombination to express a Cre recombinase under the control of the *Oprm1* promoter. In this mouse line, a cDNA encoding a functional EGFP/Cre recombinase fusion protein was inserted into exon 4 of the MOR gene, in frame and 5′ of the stop codon, as described in the studies by Gardon et al. (2014) and Erbs et al. (2015). The EGFP/Cre cDNA was generated by cloning the Cre cDNA [a gift from Daniel Metzger, Institut de Génétique et de Biologie Moléculaire et Cellulaire (IGBMC), Illkirch, France] by PCR into the BglII and EcoRI sites of the pEGFP-C2 plasmid (Clontech/Addgene), resulting in a 7 aa linker SGRTQIS between the two proteins. The cloning of Cre in 3′ in phase with EGFP and the absence of mutations were verified by DNA sequencing. The functionality of the EGFP/Cre fusion protein was verified by cotransfecting COS cells with this EGFP/Cre plasmid and with the Cre activity reporter plasmid pCMV-LneoL-Betagal (a gift from Daniel Metzger, IGBMC). Further, a T2A cleavable peptide sequence (Szymczak et al., 2004) was inserted, joining the *Oprm1* gene to the EGFP/Cre sequence, so that the EGFP/Cre enzyme is released from the receptor on translation of the MOR-T2A-EGFP/Cre fusion protein. The entire construct was verified by DNA sequencing before homologous recombination was performed. We then verified that the construct had not integrated randomly in the genome. Of note, no DNA sequencing or splicing analysis of the *Oprm1* gene was later performed in *Oprm1*-Cre mice. The genetic background of all mice was 100% C57BL/6N. Mice were group housed (maximum of five mice per cage) in a temperature- and humidity-controlled animal facility (21 ± 2°C, 45 ± 5% humidity) on a 12 h dark/light cycle with food and water available *ad libitum*. All experiments were performed in accordance with the Canadian Council of Animal Care and by the Animal Care Committees.

#### Genotyping PCR

For routine genotyping, the forward primer is ATATTATTTTCCCTGACGCGTTCTG; and the reverse primer is CTGAAGATTGACATTGTATCGAGGA. The PCR product for the *Oprm1*-Cre wild-type allele is 311 bp, and for the *Oprm1*-Cre knock-in allele is 387 bp (Extended Data Fig. 1-1).

**Figure 1.**
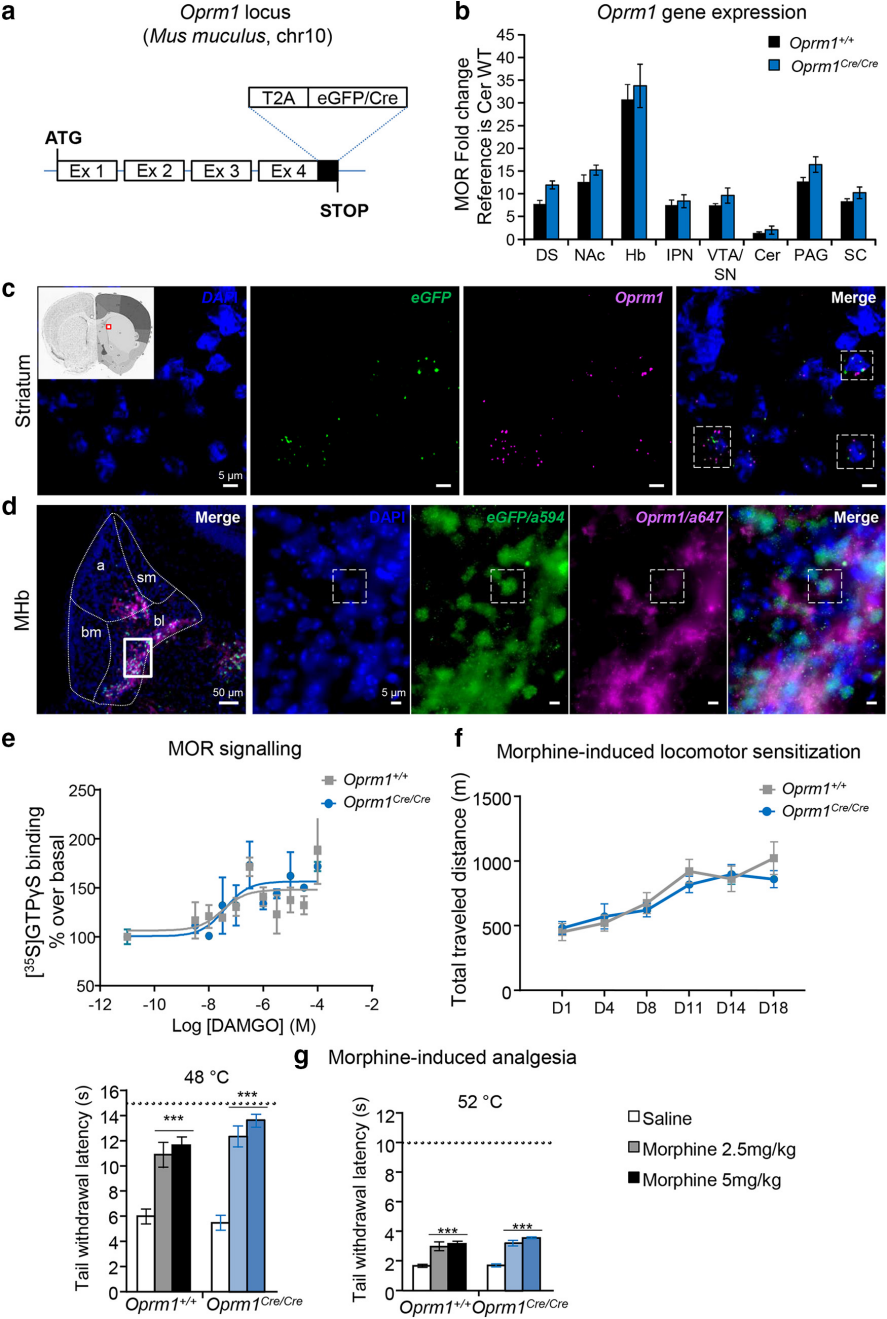
Intact MOR function in the *Oprm1*-Cre line. ***a***, Targeting strategy: a cDNA encoding a functional EGFP/Cre recombinase fusion protein is inserted in frame and upstream the stop codon of the *Oprm1* gene. In addition, a T2A cleavable peptide sequence is joining *Oprm1* gene to the EGFP/Cre sequence, so that the EGFP/Cre enzyme is released from the receptor on translation of the MOR-T2A-EGFP/Cre fusion protein (Extended Data Fig. 1-1, Knockin (KI) genotyping strategy). ***b***, *Oprm*1 mRNA expression levels are identical between *Oprm1^Cre/Cre^* and *Oprm1^+/+^* mice. Quantification was done by RT-qPCR in samples from DS, NAc, Hb, IPN, VTA/SN, Cer, PAG, and SC and shows comparable *Oprm1* transcript levels across genotypes. ***c***, Confocal imaging of RNAscope probes targeting *eGFP* (green) and *Oprm1* (purple) mRNAs in addition of DAPI staining (blue) shows colocalization of the two transcripts in dorsal striatum sections of *Oprm1^Cre/Cre^* mice (inset). Dashed lines delimit examples of MOR-EGFP/Cre-positive neurons. Magnification, 60× with immersion oil. Scale bar, 5 μm. ***d***, Left, Immunohistochemistry shows EGFP/Cre and MOR protein expression in the same habenular subdivisions. Coronal brain sections of heterozygote *Oprm1^Cre/+^* mice were stained with EGFP and MOR antibodies (Extended Data Fig. 1-2, MOR antibody validation), and fluorescence microscopy shows the expected staining mainly in basolateral (bl) and apical (a) parts of the MHb. Bm, Basomedial; sm, stria medullaris tract. Magnification:, 10×. Scale bar, 50 μm. Right, Four panels at higher magnification (inset, basolateral part of the MHb), staining reveals nuclear DAPI (blue) and EGFP/Cre (green) staining, whereas MOR staining (purple) is exclusively extranuclear. Magnification, 60× with immersion oil. Scale bar, 5 μm. Dashed lines delimit one example of an MOR-EGFP/Cre-positive neuron. ***e***, MOR signaling is preserved in *Oprm1^Cre/Cre^*mice. G-protein activation was evaluated using [^35^S]-GTPγS binding: DAMGO-induced G-protein activation is similar in striatal membranes to the two genotypes (*n* = 2 pools × 4 striatum; EC_50_, 324 nm for *Oprm1^+/+^*and 392 nm for *Oprm^Cre/Cre^*; E_max_, 148 ± 7 for *Oprm1^+/+^* and 156 ± 11 for *Oprm1^Cre/Cre^*). ***f***, Locomotor sensitization of morphine is intact in *Oprm1^Cre/Cre^*mice. Mice were injected at days 1, 4, 8, 11, 14, and 18 with morphine (40 mg/kg, i.p.). Total traveled distances recorded for 2 h are comparable in *Oprm1^+/+^*and *Oprm1^Cre/Cre^* mice (*n* = 7 animals/group). Data are presented as the mean ± SEM. ***g***, Analgesic effects of morphine are intact in *Oprm1^Cre/Cre^*mice. Analgesia was assessed by tail immersion test: identical tail withdrawal latencies were measured at 48°C and 52°C, in wild-type *Oprm1^+/+^*and *Oprm1^Cre/Cre^*mice 45 min after a single saline or morphine injection (2.5 or 5 mg/kg; *n* = 10 animals/group). Dashed horizontal lines show cutoff at 15 s for 48°C and 10 s for 52°C. Data are presented as the mean ± SEM. ***p < 0.001 morphine effect compared with saline.

10.1523/ENEURO.0433-19.2020.f1-1 Figure 1-1 KI genotyping strategy. Diagram describing the position of the primers used for genotyping. Download Figure 1-1, TIF file.

#### Dissection for mRNA and signaling testing

Mice were sacrificed by dislocation and the whole brain was quickly extracted and placed upside down in a chilled metal matrix (ASI Instruments). Cold razor blades were inserted into the 1 mm spaced coronal groove with the first most rostral one inserted at the limit of olfactory bulbs. Brain punches were dissected using 1 or 2 mm tissue corers and placed into microtubes, rapidly frozen, and stored at −80°C. A half-brain was used to prepare striatal membranes incubated with different doses of DAMGO in [^35^S]-GTPγS assay. The other half-brain was used to extract RNA in the following eight regions: dorsal striatum (DS); nucleus accumbens (NAc); habenula (Hb); interpeduncular nucleus (IPN); ventral tegmental area (VTA)/substantia nigra (SN); cerebellum (Cer); periaqueductal gray (PAG); and spinal cord (SC). The gene expression was examined by quantitative PCR.

#### Quantitative analysis of transcript expression

Quantitative real-time PCR (RT-qPCR) was adapted from the study by Meirsman et al. (2016). Six hundred nanograms of RNA was reverse transcribed using the Invitrogen M-MLV Reverse Transcriptase Kit (Thermo Fisher Scientific) according to the manufacturer instructions. The cDNA was subjected to 45 cycles of amplification using LightCycler 480 SYBR I Green Master Mix (Roche) in the LightCycler 480 II Real-Time PCR System (Roche). cDNA samples were loaded in triplicate, and a no-template control reaction, with just water, was included to check for nonspecific amplification. Relative fold changes were calculated by the comparative Ct method (2^−ΔΔCT^; Livak and Schmittgen, 2001) using B2M as a housekeeping gene.

#### mRNA *in situ* hybridization

*In situ* hybridization was performed using Advanced Cell Diagnostics RNAscope probes and reagents according to the manufacturer instruction to detect mRNA encoding MOR (*Oprm1*) and EGFP (*EGFP*). Briefly, *Oprm1^+/+^*and *Oprm1^Cre/Cre^*male mice were killed, and fresh brains were flash frozen in isopentane. The 10-μm-thick coronal sections were cut using a cryostat (Leica), directly mounted on Superfrost slides, and kept at −80°C until processing. Sections were first fixed in chilled 10% neutral buffered formalin for 15 min at 4°C, dehydrated by increasing gradient of ethanol bathes, and left to air dry for 5 min. Endogenous peroxidase activity was quenched with hydrogen peroxide reagent for 10 min, followed by protease digestion for 30 min at room temperature. The following sets of probes were then hybridized for 2 h at 40°C in a humidity-controlled oven (HybEZ II, ACDbio): *EGFP*-C1 target region 2–707 (catalog #538851, ACDbio) and *Oprm1*-C2 target region 1135–2162 (catalog #315841-C2, ACDbio). Probes for *Oprm1*, and *eGFP* were revealed using respectively Opal Dye 520 and Opal Dye 570-labeled probes. Slides were then coverslipped with Vectashield mounting medium with 4′,6′-diamidino-2-phenylindole dihydrochloride (DAPI) for nuclear staining (Vector Laboratories) and kept at 4°C until imaging.

#### [^35^S]-GTPγS binding assays

The assay was performed as previously described by several studies (Pradhan et al., 2009; Erbs et al., 2015; Meirsman et al., 2016) on membrane preparations from striatum. Striatum was dissected following mouse cervical dislocation, placed on dry ice, and stored at −80°C. To evaluate the MOR function, striatum (*n* = 2 pools × four per genotype) were pooled together and membranes were prepared by homogenizing tissues in 0.25 m sucrose with a Polytron, followed by centrifugation at 2500 rpm for 10 min at 4°C. Samples were diluted in TMEN (Tris 50 mm, MgCl2 3 mm, EGTA 0.2 mm, and NaCl 100 mm, pH 7.4) followed by an ultracentrifugation at 40,000 × *g* for 30 min at 4°C (MLA-55 rotor, Beckman Coulter). The membrane pellet was resuspended in 0.32 m sucrose by 10 strokes with a potter. Membrane preparations were diluted in 800 μl, aliquoted, and stored at −80°C. Protein concentration was determined by the Bradford assay using a standard curve of bovine serum albumin and triplicate dilution of each sample. For each [^35^S]-GTPγS binding assay, 5 μg of protein was used per well. Samples were incubated with variable concentration (3 × 10^−9^ to 2 × 10^−10^ m) of DAMGO in assay buffer containing 5 mm GDP and 0.1 nm [^35^S]-GTPγS for 1 h at 25°C. After wash and filter steps, bound radioactivity was quantified using a liquid scintillation counter (TopCount, PerkinElmer). Nonspecific binding was determined in the absence of agonist. Basal activity was determined in the presence of 10 μm GTPγS. Calculations and sigmoidal dose–response binding curves were done using GraphPad Prism 6 (GraphPad Software).

#### Tissue preparation and immunohistochemistry

Mice were anesthetized with intraperitoneal injections of 100 μl/100 g cocktail containing ketamine/xylazine/acepromazine. An intracardiac perfusion was performed with ∼10 ml of ice-cold 1× Invitrogen PBS (Thermo Fisher Scientific) pH 7.4 followed by ∼50 ml ice-cold 4% paraformaldehyde (PFA; Electron Microscopy Sciences) using a peristaltic pump at ∼10 ml/min. Brains were dissected and postfixed after 24 h at 4°C in the 4% PFA solution, cryoprotected at 4°C in 30% sucrose (Thermo Fisher Scientific) for 48 h, embedded in OCT compound (Thermo Fisher Scientific), frozen, and finally stored at −80°C. Brains were sliced into 30 μm coronal and sagittal sections using a cryostat (Leica), and sections were stored at 4°C in PBS. Immunohistochemistry was performed by washing the sections for 3 × 10 min in PBS, then for 3 × 10 min with PBS/Triton X-100 0.1% (PBS-T; Sigma-Aldrich), followed by 1 h in a blocking buffer (PBS, 3% normal donkey serum, Triton X-100 0.2%), each at room temperature with gentle agitation. Sections were incubated overnight in blocking buffer at 4°C with the following primary antibodies: 1:2000 anti-green fluorescent protein (catalog #NB100-1614, Novus; RRID:AB_523902); 1:1000 anti-MOR antibody (UMB3, catalog #ab134054, Abcam); 1:1000 anti-dsred (catalog #632496, Clontech; RRID:AB_10013483); or 1:1000 anti-tyrosine hydroxylase (catalog #ab112, Abcam; RRID:AB_297840). Sections were then washed 3 × 10 min in PBS-T, incubated for 2 h at room temperature with appropriate Alexa Fluor-conjugated secondary antibodies. Sections were washed 3 × 10 min in PBS-T with gentle agitation, placed in PBS, and mounted on to glass slides with Mowiol (PolyScience) and DAPI (0.5 μg/ml; Thermo Fisher Scientific; RRID:AB_2307445). The UMB3 MOR antibody showed no staining in sections from MOR knock-out mice (Extended Data Fig. 1-2).

#### Image acquisition

Slides were scanned on the Olympus VS120 microscope with a 10× objective. For fluorescence microscopy, an Olympus IX73 microscope with 10× or oil-immersion 60× objective was used. For confocal microscopy imaging, an Olympus FV1200 microscope with 20× or oil-immersion 60× objective, was used to take *z*-stack images.

#### Behavioral experiments

*Morphine-induced analgesia.* Mice were intraperitoneally injected with 2.5 or 5 mg/kg morphine or saline, and analgesia was tested using tail immersion (Erbs et al., 2015). Briefly, the mouse was maintained in a cylinder and the tail was immersed in a water bath set at 48°C with a cutoff time of 15 s. Mice were allowed to recover for 1 min followed by tail immersion in a second water bath set at 52°C with a cutoff time of 10 s. The baseline response was measured for tail flick before the first injection, and the morphine-induced analgesia tests were performed 45 min after the injection.

*Morphine-induced locomotor sensitization.* Locomotor activity was measured in Plexiglas activity boxes (20 × 20 × 20 cm) surrounded by horizontal and vertical infrared sensor beams, assisted by VersaMax software. To evaluate the morphine-induced hyperlocomotion at the first session, mice were allowed to explore the boxes for 1 h. Mice were then injected with saline (1 ml/kg) and returned to the boxes for 1 h followed by an intraperitoneal injection of morphine (40 mg/kg) or saline, and activity was recorded for 2 h. For locomotor sensitization, mice were injected twice per week with saline and placed in the boxes for 1 h followed by morphine (40 mg/kg) injection and activity was recorded for 2 h. Measurements were taken on days 1, 4, 8, 11, 14, and 18, as adapted from the study by Darcq et al. (2012).

*Stereotaxic surgery.* Animals were anesthetized with 5% isoflurane for 5 min and maintained at 2% isoflurane. For viral Cre recombination, adult *Oprm1^Cre/Cre^*male mice were injected unilaterally with 100 nl of AAV2.EF1a.DIO.mCherry (RRID:Addgene_20299) in the medial Hb [MHb; anteroposterior (AP), −1.35 mm; mediolateral (ML), −0.25 mm; dorsoventral (DV), −2.8 mm]. For optogenetic experiments, adult *Oprm1^Cre/Cre^*male mice were injected unilaterally with 400 nL of AAV2.EF1a.DIO.ChR2-mCherry (RRID:Addgene_20297) or AAV2.EF1a.DIO.mCherry in the VTA (AP, −3.3 mm; ML, −0.5 mm; DV, −4.3 mm). Two weeks after virus injection, fiber-optic ferrules (200 μm; numerical aperture, 0.37) were implanted above the VTA (AP, −3.3 mm; ML, −0.5 mm; DV, −4.1 mm). The implant was secured using a first layer of Metabond followed by a layer of dental cement. Mice were allowed to recover for at least 4 weeks after infusion of virus before habituation to the optic cord and behavioral testing. The injected and implanted mice are designated as *Oprm1*-Cre^VTA-VTA::ChR2^.

*Real-time place preference.* Five weeks after the viral injections, mice were placed in a custom behavioral arena (black Plexiglas, 50 × 50 × 25 cm) divided into two identical chambers and allowed to explore each of the two chambers for 20 min. Using an Anymaze hardware controller connected to the laser, light stimulation (473 nm, 10 mW) at 0, 10, 20, or 40 Hz (10 ms pulse width) was delivered through fiber-optic implants during the duration of their time spent in the light stimulation chamber. Mice received no light stimulation in the “no stimulation” chamber. At the start of the session, the mouse was placed in the nonstimulated side of the chamber. The percentage of time spent on the paired stimulation side was recorded via a CCD camera interfaced with the Anymaze (Stoelting) software.

#### Statistical analysis

All data are presented as the mean SEM. Statistical analysis was assessed using *t* tests or repeated-measures ANOVA. When ANOVA reached significance, a Tukey’s HSD test was conducted. Nonsignificance was defined as *p* > 0.05, and significance as *p < 0.05, **p < 0.01, and ***p < 0.001.

## Results

Unsuccessful attempts to develop transgenic mouse lines using both short and bacterial artificial chromosome promoters of the mu-opioid receptor gene (*Oprm1*), led us to use homologous recombination to insert the Cre recombinase gene into the *Oprm1* gene locus. A knock-in strategy for the *Oprm1* gene was designed (Fig. 1*a*) to generate a large precursor protein, which would be further cleaved to release both the native MOR protein and a functional EGFP/Cre recombinase in cells that normally express the MOR. This approach produced the *Oprm1^Cre/Cre^*(or *Oprm1*-Cre) mouse line, which we characterized extensively.

**Figure 2.**
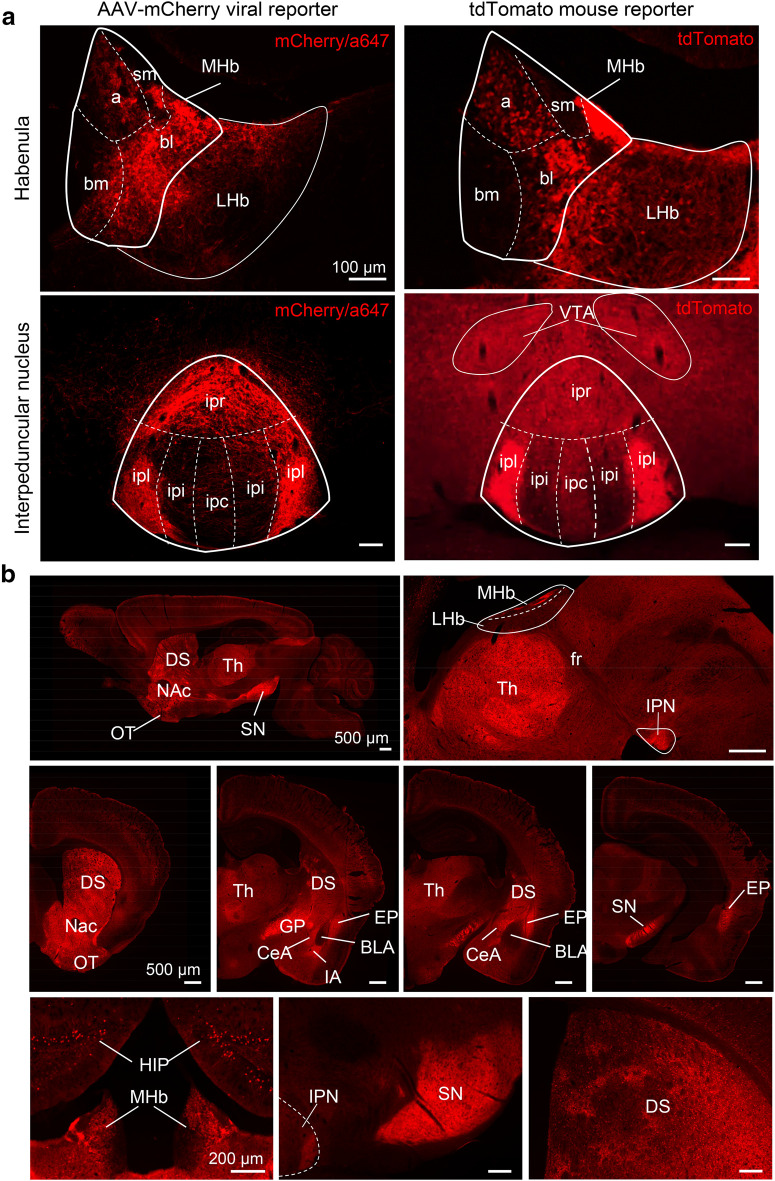
Cre-mediated recombination is efficient in the *Oprm1*-Cre line. ***a***, Cre-mediated expression of the fluorescent reporter tdTomato in the habenula–interpeduncular pathway. Left, Cre recombination on local injection of an AAV2.EF1a.DIO.mCherry viral reporter in the MHb; right, Cre recombination on breeding Cre-dependent tdTomato reporter mice with the *Oprm1*-Cre line both lead to a strong fluorescent signal apical (a) and basolateral (bl) of the MHb, as well as in the rostral (ipr) and lateral (ipl) areas of the IPN. Scale bar, 100 μm. ***b***, Cre-mediated tdTomato expression in the adult brain of *Oprm1*-Cre × tdTomato mice. Top, Whole-brain sagittal views show high tdTomato expression in the thalamus, as well as the entire mesolimbic (left) and MHb-IPN (right) pathways. Scale bar, 500 μm. Middle, Coronal sections show strong fluorescence in the basal ganglia and thalamus, as well as in most amygdalar nuclei with the notable exception of basolateral amygdala. Scale bar, 500 μm. Bottom, Higher-magnification highlights both cell bodies and/or fibers patterns of labeled neurons. Scale bar, 200 μm. Slides were scanned on the Olympus VS120 with a 10× objective. BLA, Basolateral amygdala; CeA, central amygdala; EP, endopiriform nucleus; fr, fasciculus retroflexus; GP, globus pallidus; HIP, hippocampus; IA, intercalated amygdala; LHb, lateral Hb; OT, olfactory tubercle; Th, thalamus.

10.1523/ENEURO.0433-19.2020.f1-2 Figure 1-2 UMB3 expression and antibody validation. Coronal sections of habenula were stained with UMB3 antibody and show MOR expression in wild-type mice (left), but no signal could be detected in the MOR KO mice (right). Scale bar, 100 μm. Download Figure 1-2, TIF file.

We first tested whether the insertion of the T2A-EGFP/Cre cDNA into the *Oprm1* gene locus modifies levels of gene transcription. Quantitative mRNA analysis in several brain regions, including most MOR-enriched regions, revealed that the genomic modification does not disrupt *Oprm1* transcription (Fig. 1*b*). Using EGFP primers, we also detected the *egfp* transcript in *Oprm1^Cre/Cre^* but not *Oprm1*^+/+^ control mice, suggesting that the entire *Oprm1-T2A-eGFP/Cre* transcript is transcribed in the knock-in line (data not shown). We further performed double *in situ* hybridization using separate probes for *Oprm1* and *eGFP/Cre* mRNAs in brain sections from *Oprm1^Cre/Cre^* mice (two mice, eight sections) and found colocalization of the two transcripts in all the sections examined (Fig. 1*c*, level of striatum).

Second, we examined expression of MOR and EGFP/Cre proteins using immunohistochemistry in brain sections from heterozygous *Oprm1^Cre/+^* mice. In the MHb (i.e., the best MOR-enriched region), we observed predominant expression of the two proteins in both basolateral and apical subregions (Fig. 1*d*, left) as expected (Gardon et al., 2014). Higher magnification (Fig. 1*d*, right panels) showed distinct subcellular distribution of the two proteins, as expected from the natural localization of MOR (cytoplasm/membrane) and EGFP/Cre (nuclear). The latter observation suggests that the T2A-EGFP/Cre fusion protein was mostly cleaved, which was further supported by Western blot (data not shown) and the expected activity for the two proteins (see below).

Third, we investigated whether MOR function is intact in this mouse line. We tested MOR signaling in response to DAMGO, a MOR-selective agonist. The [^35^S]-GTPγS binding assay showed similar potency and efficacy of DAMGO in samples from the two genotypes, suggesting that MOR-mediated G-protein activation is intact in the *Oprm1*-Cre line (Fig. 1*e*). Next, we compared the two best documented *in vivo* effects of morphine in *Oprm1^Cre/Cre^*and *Oprm1^+/+^*mice. Morphine-induced hyperlocomotion, as well as locomotor sensitization were measured in *Oprm1^Cre/Cre^* and *Oprm1^+/+^*mice (Fig. 1*f*). A two-way, mixed-design ANOVA, with genotype as a between-subjects factor, and days since injection as a within-subjects factor, indicated a main effect only for days (*F*_(5,60)_ = 35.19, *p* < 0.001). The interaction term was not significant (*F*_(5,60)_ = 1.70, *p* > 0.05). Pairwise comparisons tests were performed using a Tukey’s HSD test. The mean total traveled distance at days 11, 14, and 18 was significantly higher compared with days 1, 4, and 8 (*p* < 0.001), and day 8 was significantly higher than day 1 (*p* < 0.05). Morphine therefore induced similar hyperlocomotion and sensitization in the two genotypes. Morphine analgesia was assessed using the tail immersion test (Fig. 1*g*). At 48°C, a two-factor (genotype × injection treatment) between-subject ANOVA yielded significant main effects for injection treatment (*F*_(2,54)_ > 53.77, *p* < 0.0001). The interaction term was not significant (*F*_(2,54)_ = 1.724, *p* > 0.05). Tukey’s HSD tests computed on the main effect for treatment indicated that the mean tail withdrawal latencies were significantly increased for both genotypes and at 2.5 and 5 mg/kg morphine doses (all *p* values < 0.001). Similarly at 52°C, a two-factor (genotype × injection treatment) between-subject ANOVA yielded a significant main effect for the treatment (*F*_(2,51)_ > 45.32, *p* < 0.0001), with no significant interaction (*F*_(2,51)_ = 0.453, *p* > 0.05). Tukey’s HSD tests indicated that mean tail withdrawal latencies were significantly increased for the two genotypes and at the two morphine doses (all *p* values < 0.001). Together, results show intact morphine effects on both activity and pain perception in mutant mice, and overall the data suggest that MOR signaling and function *in vivo* are maintained in the *Oprm1-*Cre line.

Fourth, we examined whether the EGFP/Cre fusion protein is able to mediate Cre/LoxP recombination in the *Oprm1*-Cre line. Of note, although the EGFP/Cre fusion has the advantage of allowing detection of the recombinase, non-EGFP fluorophores should be used when combining with other reporters. We therefore used red fluorescent reporters in the subsequent experiments. We first injected a Cre-dependent fluorescent reporter AAV2-mCherry virus in the MHb, and found a strong fluorescent signal (Fig. 2*a*, left) with a pattern similar to the EGFP/Cre signal (Fig. 1*c*) in apical and basolateral MHb. Several compartments of the IPN were also strongly labeled, indicating that the fluorescent reporter is transported along fibers to the major projection site (Fig. 2*a*, left), as expected (Gardon et al., 2014). To get a brain-wide view of Cre-mediated recombination in the *Oprm1*-Cre line, we crossed the mice with Cre-dependent reporter Rosa*^lsltdTomato^* mice (see Materials and Methods and Movie 1). The tdTomato protein was strongly expressed in MHb, fasciculus retroflexus, and IPN (Fig. 2*a*, right**)**, with a pattern similar to that observed on Cre-dependent viral reporter expression except that areas out of the MHb–IPN pathway were also labeled. In particular, fluorescence was observed throughout the mesolimbic pathway, including striatal patches, direct pathway projections neurons, and the VTA/SN that are typical of MOR expression (Cui et al., 2014). Recombination also occurred in the central amgydala, intercalated amygdala, and the endopiriform nucleus, but not in the basolateral amygdala, concordant with sites of high MOR expression (Erbs et al., 2015). Together, these anatomic data demonstrate that the EGFP/Cre protein is functional. Further, Cre activity is found at known sites of MOR expression, indicating accurate transcriptional control of *Cre-egfp* under the *Oprm1* promoter. Staining patterns observed here differ from those previously reported in MOR-mCherry mice (Gardon et al., 2014; Erbs et al., 2015), as two distinct knock-in strategies were used to label either MOR-expressing cells (MOR-Cre mice; this study) or the receptor itself (MOR-mCherry mice).

Movie 1. 3D views of the whole brain. 10.1523/ENEURO.0433-19.2020.video.1

**Figure 3.**
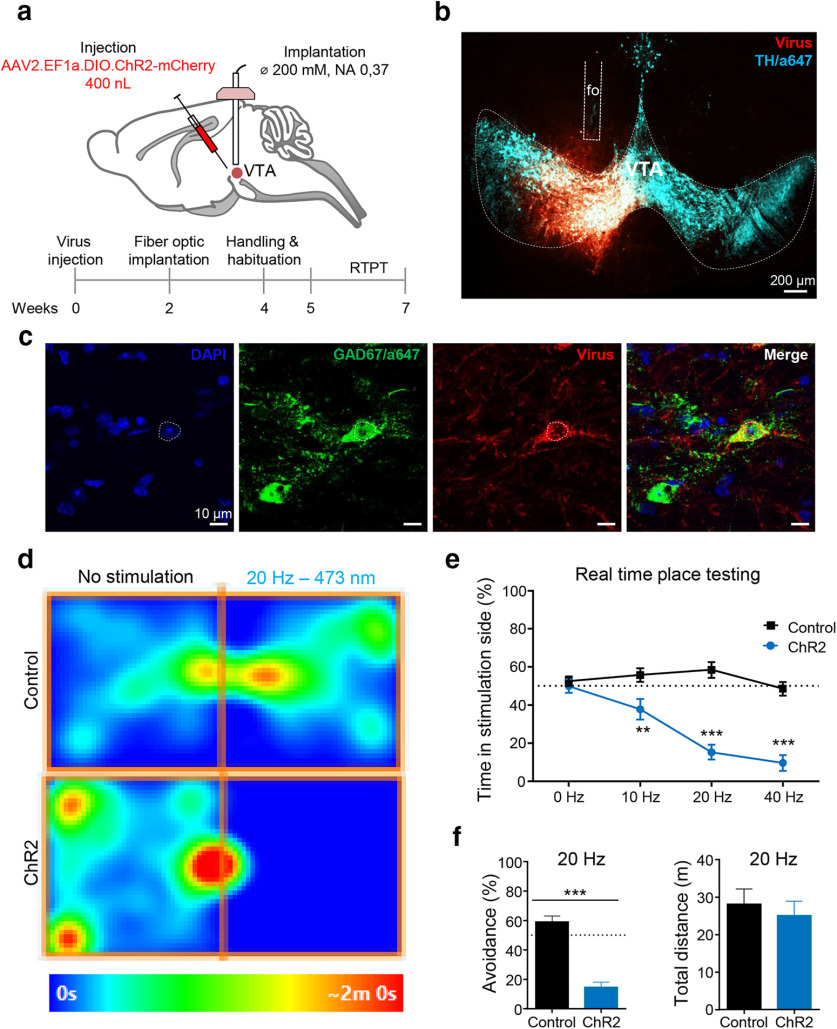
The *Oprm1*-Cre line is amenable to optogenetics. *Oprm1*-Cre^VTA-VTA::ChR2^ photostimulation causes behavioral avoidance. ***a***, Diagram showing viral delivery of AAV2.EF1a.DIO.ChR2-mCherry (channelorhodopsin) or AAV2.EF1a.DIO.mCherry (control) into the VTA and fiber-optic implantation above the VTA, a well as a timeline for the experimental procedure. ***b***, Representative image of viral expression (red), optic fiber implantation, and VTA dopamine cells immunostained with anti-tyrosine hydroxylase (TH)/Alexa Fluor 647 (blue). Tissues were observed on an inverted epifluorescence microscope. ***c***, Confocal imaging of VTA sections stained with GABA antibody show overlapping with viral expression. Scale bar, 10 μm. ***d***, Occupancy plots for representative individual at 20 Hz. Mice were free to explore the two- chamber RTPT apparatus for 20 min. Mice then received a blue stimulation when entering the light-paired side at 0, 10, 20, and 40 Hz (473 nm, 10 mW, 10 ms pulse width) on 4 consecutive days, as described by Seo et al. (2016). ***e***, Activation of VTA MOR neurons produces place avoidance. Light stimulation in *Oprm1*-Cre^VTA-VTA::ChR2^ mice induced significant behavioral avoidance to the light-paired side compared with control group (*n* = 7–8/group). The graph shows frequency responses of mice receiving a blue stimulation when entering the light-paired side (473 nm, 10 mW, 10 ms pulse width at 0, 10, 20, and 40 Hz; *n* = 9–10, control vs ChR2). Data are expressed as the mean ± SEM. **p < 0.01, ***p < 0.001. ***f***, Further analysis of the 20 Hz simulation condition indicated significant avoidance for the light-paired side without affecting the total distance traveled (*p* > 0.05).

Finally, we tested whether *Oprm1^Cre/Cre^*mice can be used for cell-specific optogenetic manipulation of MOR neurons. Earlier studies have demonstrated that optical stimulation of GABAergic interneurons in the VTA induces avoidance behavior, as a consequence of dopamine (DA) neuron inhibition (Tan et al., 2012). MOR is a G_i_-coupled receptor expressed in these interneurons, and a best known mechanism for MOR-mediated reward is through the inhibition of these VTA GABA interneurons, an activity that in turn disinhibits DA neurons (Johnson and North, 1992; Fields and Margolis, 2015). We therefore hypothesized that optogenetic activation of MOR-EGFP/Cre-expressing neurons in the VTA of *Oprm1^Cre/Cre^*mice would produce a place avoidance, as did the stimulation of the entire population of VTA GABAergic neurons in the study by Tan et al. (2012). We injected a Cre-dependent AAV2-channelrhodopsin virus in the VTA (Fig. 3*a*,*b*) to express ChR2 mainly in MOR/GABAergic interneurons (Fig. 3*c*) and tested animals using a real-time place-testing (RTPT) paradigm (Fig. 3*d*). A two-way ANOVA with virus as a between-subject factor and frequency as a within-subject factor yielded a significant interaction (*F*_(3,51)_ = 16.93, *p* < 0.001). Simple effect test revealed that the *Oprm1*-Cre^VTA-VTA::ChR2^ mice spent significantly less time on the side paired with light stimulation than control mice at 10 Hz (**p < 0.01), 20 Hz, and 40 Hz (***p < 0.001) compared with control mice (Fig. 3*d*,*e*), indicating that optical stimulation triggered place avoidance. The stimulation did not affect total activity measured at 20 Hz (unpaired *t* test, *t*_(14)_ = 1.497, *p* > 0.05; Fig. 3*f*). Together, data demonstrate that the activation of MOR-positive neurons in the VTA produce avoidance, as predicted. The *Oprm1*-Cre line, therefore, efficiently drives Cre-mediated recombination to modulate behavior.

## Discussion

Originally developed to allow gene targeting in specific cells using Cre/LoxP recombination (Gavériaux-Ruff and Kieffer, 2007), Cre driver lines are also extensively used to label specific neuronal populations and visualize their connectivity patterns when combined with Cre-dependent fluorescent reporter mouse lines or viruses (Cai et al., 2013; Soden et al., 2014), or to monitor the activity of phenotypically defined neuronal ensembles using Cre-dependent calcium indicators (Russell, 2011). The utility of mouse Cre lines has further expanded with the advent of optogenetic and chemogenetic approaches, which allow manipulating Cre-expressing neurons to understand the circuit mechanisms underlying behavior (Fenno et al., 2011; Sternson and Roth, 2014). Transgenic Cre lines have now been generated by individual laboratories, as well as large initiatives, including the GENSAT program (Gong et al., 2007) the NIH Blueprint Cre driver Network (Taniguchi et al., 2011) and the Allen Brain Institute.

Lines providing access to the opioidergic circuitry remain limited (for review, see Darcq and Kieffer, 2018), and targeting cells responding to medicinal and abused opioid drugs is a desirable goal. To study opioid peptide-expressing cells, Cre lines using *Pdyn*, *Penk*, and *Pomc* gene promoters have been created (Harris et al., 2014, for *Penk* and *Pomc* lines), and *Pdyn*-Cre mice were used to characterize subpopulations of striatal neurons in the direct pathway (Al-Hasani et al., 2015) study D_1_/D_2_-type neuron activity balance in the nucleus accumbens (Tejeda et al., 2017), rescue MOR expression in the striatum (Cui et al., 2014), or investigate amygdala circuitry (Crowley et al., 2016). On the receptor side, one knock-in Cre line was created to gain genetic access to kappa-opioid receptor (KOR)-expressing cells (Cai et al., 2016) and used to study peripheral KOR neuron terminals in pain control (Snyder et al., 2018). This work provides a tool to access MOR neurons.

Full characterization of this novel *Oprm1*-Cre driver line demonstrates the following: (1) MOR signaling and function are preserved, as shown by intact G-protein activation and normal morphine-induced analgesia, locomotor stimulation, and sensitization; (2) EGFP/Cre is detectable, and the expression pattern matches MOR expression; and (3) the EGFP/Cre recombinase is functional and effectively drives both the expression of Cre-dependent reporter genes and optogenetic sensors in MOR-expressing neurons. Together with a recently published inducible MOR-CreER mouse line (Okunomiya et al., 2020), this *Oprm1*-Cre line is a unique tool for both mapping and functional studies of MOR-positive neurons, and will be of broad interest for opioid, pain, reward, and addiction research.

## Synthesis

Reviewing Editor: Yavin Shaham, NIDA-IRP/NIH Decisions are customarily a result of the Reviewing Editor and the peer reviewers coming together and discussing their recommendations until a consensus is reached. When revisions are invited, a fact-based synthesis statement explaining their decision and outlining what is needed to prepare a revision will be listed below. The following reviewer(s) agreed to reveal their identity: Brandon Harvey, Robert Messing.

Synthesis Statement for Author (Required):

The reviewers found significant merit in the study but have raised several concerns that should be addressed in the revision. I enclose below the individual comments of the reviewers and our consensus recommendation of how to address them. In addition to their comments, also address the comments below:

1. Methods: please add RRID number for the different antibodies and other reagents when relevant

2. Results: please move formal statistical results (F and p values) from the figure legends to the Results section

3. Page 7: change ‘rev in’ to ‘reviewed in’

4. Discussion: the optogenetic data showing avoidance behavior after stimulation of MOR in VTA contradict many studies of Roy Wise, Satoshi Ikemoto, Jane Stewart, and others showing that injections of MOR agonists into the VTA are rewarding and reinstate drug seeking in rats, presumably by increasing dopamine release in nucleus accumbens. Please briefly discuss this literature and potential reasons for the discrepant results.

REVIEWER 1

his method/reagent paper describes the construction and characterization of a transgenic mouse engineered to express a GFP-Cre recombinase fusion protein from the mu opioid receptor (Oprm-1) locus. They employ a T2A self cleaving peptide sequence to express two proteins from single RNA made from the Oprm-1 promoter. Evidence is presented that homozygous Cre-GFP animals show similar phenotype to WT animals based on analgesia response, Opr1m1 gene expression, MOR signaling and morphine induced locomotor sensitization. The animal is used to selectively express channel rhodopsin in VTA MOR+ cells and demonstrate optogenetic manipulation to achieve an established avoidance behavior. There are no novel findings using the newly made and characterized mouse. Overall, the authors do a diligent job of characterizing the animals, but before these mice are released into the scientific wild as a powerful tool for studying opioids and pain, there are few other points for consideration that are outlined below.

It appears in Figure 1C that there is very little colocalization of eGFP and MOR or is that high background fluorescence? Does the Cre_GFP have an NLS? It appears there is some overlap of MOR/eGFP in “cytosol” of Figure 1C second panel suggesting there may be some GFP still fused to MOR (which may decrease the immunoreactivity of GFP depending on where GFP antibody epitope(s) is located). Given the MOR antibody used in the study for immunostaining can be also be used for western blots, the authors should consider examining MOR by western blot to see what relative proportion may be still coupled to Cre-GFP as the T2A is not 100% effective.

Editor: please consider adding western blot data to the revision to address the reviewer’s concern

Was copy number performed for transgene to ensure additional (random) integration of the transgene did not occur?

Editor: please add this information to the revision

The MOR mRNA undergoes alternative splicing that can influence its signaling and other cell biology. Does the presence of the T2A-Cre-GFP alter splicing? This could affect opioid response in the mouse. See Bolan et al Synapse 2004; Doyle et al. Gene 2007; Puig and Gutstein JCI 2017, as some examples of the importance of splicing in MOR biology.

Editor: please address this issue in the revision with either additional data or text changes

Minor Points

Title overstates findings. There are three opioid receptors so the animal won’t necessarily target all opioid responsive neurons as title implies. The title could be restricted to the generation and characterization of a transgenic mouse coexpressing Cre from the endogenous mu opioid receptor locus. “Knock-In mu opioid receptor-Cre” could be a construct “knocked in” to another locus e.g. Rosa26. Consider revising title.

Editor: we recommend changing the title accordingly

Has UMB3 antibody for MOR been validated in KO background? Although there are numerous references, it would be useful to cite that it was validated at some point.

Editor: Please add this information to the revision

For the genotyping assay, it would be helpful to indicated on the transgene schematic where the genotyping primers bind.

Editor: Please add this information to the revision

Was any sequence analysis conducted through the homologous arm regions to ensure no mutations introduced following recombination of the transgene into the Oprm-1 site?

Editor: Please add this information to the revision

The inclusion of GFP creates some limitations in the utility of the mouse by eliminating the reporters/tags/secondary antibodies that require the same ex/em spectra as GFP. This caveat should be discussed and indicated as a limitation with this mouse. The positive aspects of having GFP should also be highlighted.

Editor: Please address this comment in the revised Discussion

REVIEWER 2

In this paper the authors present a new mu opioid receptor Cre mouse. This mouse line that could be very useful for many labs who study opioid circuitry and behavior. Its utility is critically dependent on the fidelity of Cre expression with MOR. I have the following comments:

1. The language needs to be edited and improved. It is quite rough in the methods and could use improvement in the introduction as well.

Editor: Please make text changes accordingly

2. Figure 1c: It seems that there is Cre-EGFP expression in areas that do not express MOR. Individual panels depicting MOR and EGFP fluorescence should be included in addition to the merged images. Higher resolution images are needed of brain regions with high MOR expression like the medial habenula, NAc and dorsal striatum (where MOR’s have been historically thought to localize to striosomes) and the intercalated nucleus of the amygdala.

Editor: please add new images to address the reviewer’s concerns. Also discuss this concern in the revised Discussion

3. Figure 2 shows several images resulting from viral expression of a Cre dependent reporter or from crossing the Cre mouse with a reporter mouse. Unless one is quite familiar with the pattern of MOR expression it is difficult to decide whether the MOR-Cre mouse recapitulates this pattern. Why not show overlays with eGFP, which would help in differentiating between cell body and fiber fluorescence?

Editor: we recommend showing the data as suggested by the reviewer

4. Figure 3, panel b: There seems to be ectopic expression of CHR2 outside the VTA unless the staining outside the VTA corresponds to fibers. It would be helpful if the authors performed in situ hybridization to determine if MOR mRNA expression is restricted to GABAergic neurons in the VTA.

Editor: These additional can be added but they are not mandatory for a successful revision. Please address the issue of potential ectopic expression in the revision

5. There also seem to be discrepancies in the expression pattern in the MOR Cre mice described in this paper and the MOR-mCherry mouse (described in Gardon et al., Neuroscience 2014 and Erbs et al Brain Struc Func 2015), especially the notable absence of major fluorescence in the thalamus in MOR-mCherry mice. Major discrepancies between the two lines should be discussed in the paper.

Editor: please address this issue in the revised Discussion
