# Supplementary figures and images for: Targeting Morphine-Responsive Neurons: Generation of a Knock-In Mouse Line Expressing Cre Recombinase from the Mu-Opioid Receptor Gene Locus
Source: eNeuro. 2020 May 29;7(3):ENEURO.0433-19.2020. doi: 10.1523/ENEURO.0433-19.2020 (PMC7266138; doi:10.1523/ENEURO.0433-19.2020)

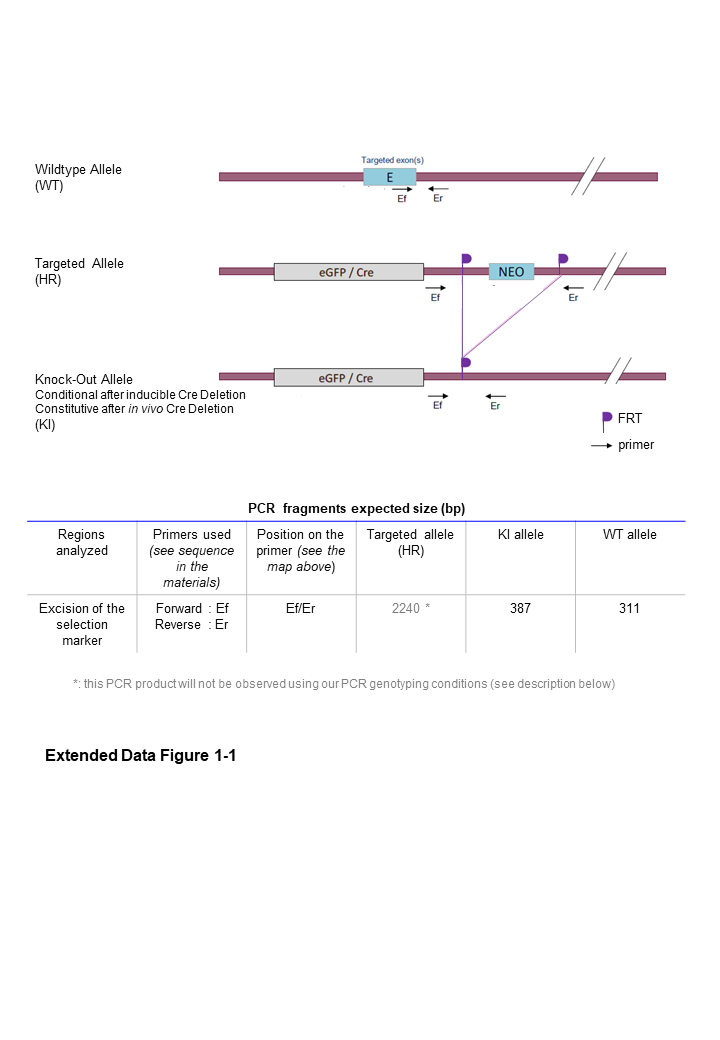

Supplement: Figure 1-1 — KI genotyping strategy. Diagram describing the position of the primers used for genotyping. Download Figure 1-1, TIF file. [file enu-eN-MNT-0433-19-s02.tif]

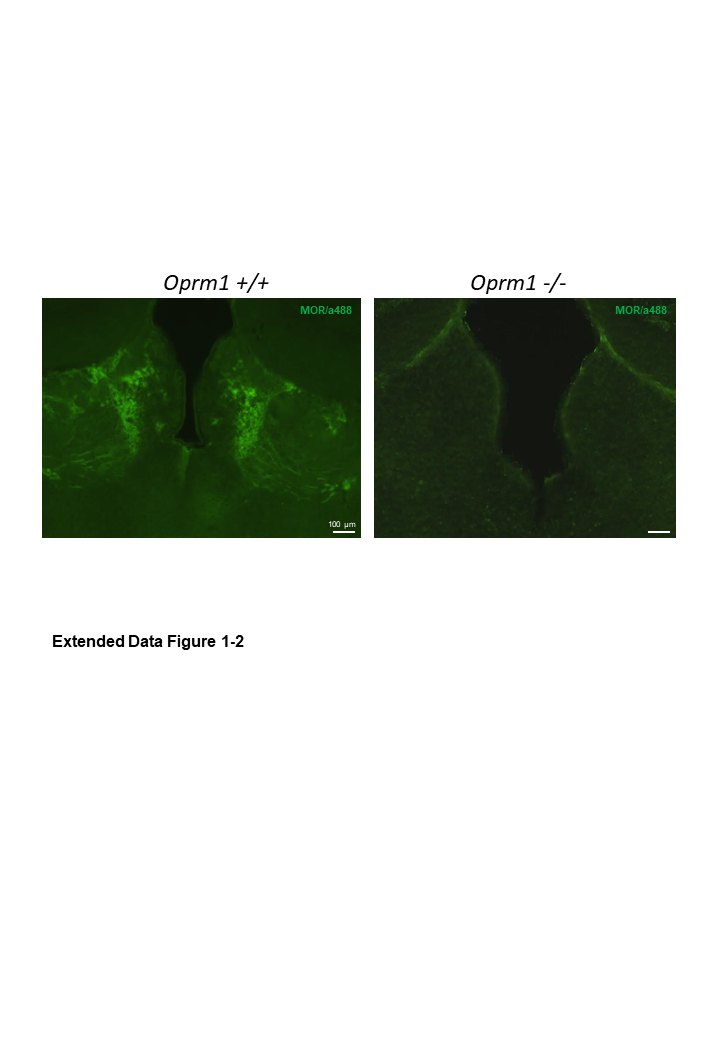

Supplement: Figure 1-2 — UMB3 expression and antibody validation. Coronal sections of habenula were stained with UMB3 antibody and show MOR expression in wild-type mice (left), but no signal could be detected in the MOR KO mice (right). Scale bar, 100 μm. Download Figure 1-2, TIF file. [file enu-eN-MNT-0433-19-s03.tif]
